# Case Series: The Coexistence of Thrombocytopenia and Thromboembolism in COVID-19 Patients on ECMO: A Case Series and Literature Review

**DOI:** 10.3389/fmed.2021.731352

**Published:** 2021-09-08

**Authors:** Can Jones, Kai Chen, Vijay Narendran

**Affiliations:** ^1^John Fitzgerald Kennedy Medical Center Palm Beach Regional Graduate Medical Education Consortium, Atlantis, University of Miami, Miami, FL, United States; ^2^John Fitzgerald Kennedy Medical Center, Atlantis, Miami, FL, United States

**Keywords:** thrombocytopenia, thromboembolism, COVID-19, ECMO, anticoagulation

## Abstract

Thrombocytopenia and thromboembolism are common complications in coronavirus disease 2019 (COVID-19) patients. The fact that COVID-19 patients develop both thrombocytopenia and thromboembolism has been observed, and multiple studies have investigated the underlying pathophysiology. Extracorporeal membrane oxygenation (ECMO) is reserved for COVID-19 patients who develop respiratory failure and not respond to conventional mechanical ventilation. ECMO induces thromboembolism and raises the incidence of developing thromboembolic events in COVID-19 patients. Here, we report the hospital courses and outcomes of three COVID-19 patients who were treated with ECMO, then developed both thrombocytopenia and thromboembolism. The coexistence of thrombocytopenia and thromboembolism challenges the clinical treatment strategy, including the decision of initiating anticoagulation. Based on current data, anticoagulation is recommended to all hospitalized COVID-19 patients unless there is active bleeding, previous bleeding history within 3 days, or platelet count is lower than 30,000 cells/μl. Further investigation into the mechanisms and implications of thrombocytopenia and thromboembolism in patients with COVID-19 pneumonia will lead to significantly improved outcomes and prognosis for the patients.

## Introduction

Thrombocytopenia and thromboembolism are common complications in coronavirus disease 2019 (COVID-19) patients.

The possible mechanisms (1) of thrombocytopenia in COVID-19 patients include decreased platelet production and increased platelet destruction and consumption. COVID-19-induced cytokine storm leads to the destruction of bone marrow progenitor cells and causes decreased platelet production; direct infection of hematopoietic and bone marrow stromal cells also has a consequence of bone marrow suppression. Immune thrombocytopenia has been frequently reported in COVID-19 patients. Increased of autoantibodies and immune complexes lead to platelet destruction (2). Pulmonary endothelial injury from COVID-19 infection triggers platelet activation, aggregation, and formation of microthrombi, which causes increased platelet consumption. Meanwhile, acute respiratory distress syndrome (ARDS) secondary to COVID-19 pneumonia contributes to dysfunction of megakaryopoiesis, since the lungs are important sites to release platelets from mature megakaryopoiesis, which leads to delayed phase thrombocytopenia (3). According to current studies, thrombocytopenia indicates poor prognosis (4) and high mortality of hospitalized COVID-19 patients (5).

Interestingly, COVID-19 patients tend to develop thromboembolism, which often leads to sudden deterioration and death. D-dimer is an excellent marker to monitor hypercoagulability and predict outcome (6). Evidence suggests that there are multiple mechanisms involved in the development of thromboembolism. Endothelial dysfunction plays a critical role in the pathogenesis (7). Endothelial injury precipitates platelet activation and adhesion, leukocyte aggregation, cytokine storm, and complement activation. The expression of pro-inflammatory cytokines are significantly elevated in COVID-19 patients, and cytokine storm triggers coagulation activation and thrombin generation (8). Interleukin 6 (IL-6) is considered a dominant inflammatory cytokine by activating coagulation pathway, stimulating megakaryopoiesis, and facilitating the production of coagulation factors. IL-6 has been investigated as a promising immunotherapy target for COVID-19 infection (9). Complement activation is also thought to trigger the formation of systemic thrombus through recruiting inflammatory cytokines and possible complement-mediated thrombotic microangiopathy (7, 8). Recent study revealed that spike surface glycoprotein expressed by the virus could bind to angiotensin-converting enzyme 2 (ACE2), decrease its expression, and stimulate renin-angiotensin system (RAS). This process mediates platelet activation and adhesion, eventually promoting systemic thromboembolism (9).

Patients on extracorporeal membrane oxygenation (ECMO) are at risk of developing thromboembolism. Thrombus formation within the extracorporeal circuit is the main reason that leads to systemic thromboembolism, including formation of pulmonary embolism (PE). The mechanism is possibly that by contacting blood and non-endothelial surfaces, ECMO triggers activation of coagulation pathway and inflammatory response (10). Thrombocytopenia is often complicated in ECMO as well due to shearing force in circuit and heparin-induced thrombocytopenia (HIT). However, there is no valid screening score which could be used specifically in ECMO patients for HIT prior to thromboembolic events (11).

The balance between bleeding prevention and thromboembolic prophylaxis in the setting of coexistence of thrombocytopenia and thromboembolism in COVID-19 patients is critical. Here, we report three COVID-19 patients who were treated with ECMO-developed thromboembolism and thrombocytopenia. HIT was excluded from all of the cases.

## Methods

**Figure 1:**
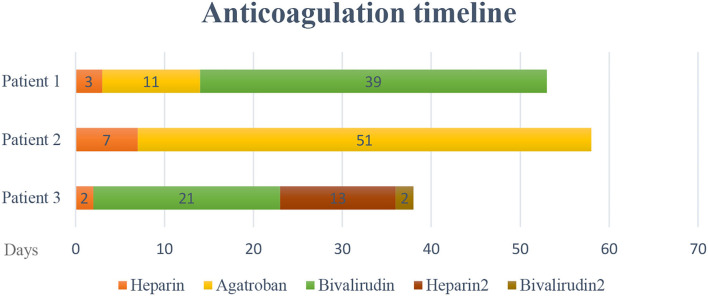
Anticoagulation course of thromboprophylaxis and thromboembolism treatment.

**Figure 2:**
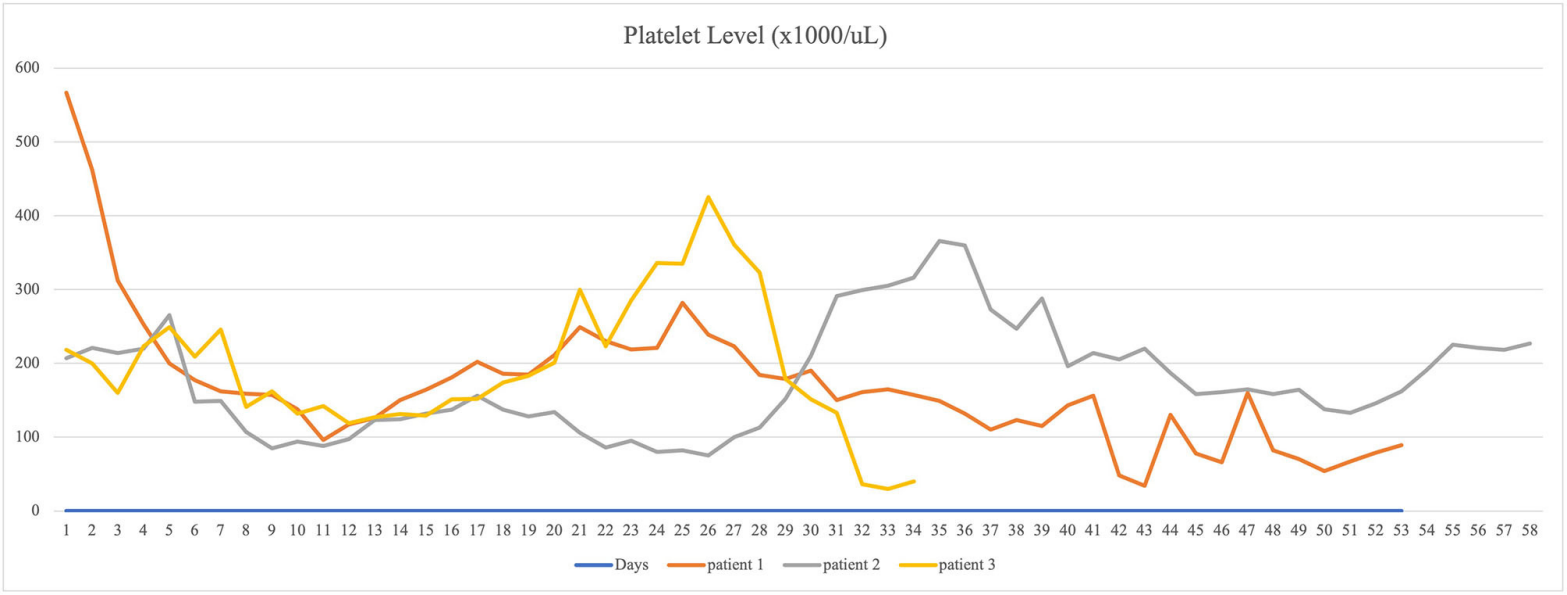
The course of platelet counts of the three ECMO-managed COVID-19 patients.

The study was approved by the local institutional review board. Data of three patients who were diagnosed with COVID-19 pneumonia by polymerase chain reaction (PCR) and managed with ECMO were collected (Tables 1, 2). Anticoagulation protocol was initiated on hospital day one. All these three patients developed thrombocytopenia and thromboembolism while on ECMO support (Figures 1, 2). CARE guideline was followed throughout the study.

**Table 1:** Clinical characteristics of three patients on admission.

**Patient**	**1**	**2**	**3**
Age (years)	35	51	52
Gender	Male	Male	Male
Race	Black	Black	Hispanic
Body mass index (BMI)	20.9	22.1	27.6
Hypertension	Yes	Yes	No
Diabetes	Yes	Yes	No
Hyperlipidemia	No	No	No
Chronic lung disease	No	No	No
Chronic kidney disease	No	No	No
Malignancy	No	No	No
Active smoker	No	No	No
Using ACEI/ARB	No	No	No

**Table 2:** Clinical presentations and baseline laboratory studies on admission.

	**Patient 1**	**Patient 2**	**Patient 3**
COVID-19 symptoms	Fatigue, fever, cough, and abdominal pain	Fever and dyspnea	Fever and dyspnea
**Vital signs on admission**			
Heart rate (bpm)	133	110	58
Respiratory rate (bpm)	28	21	28
Blood pressure (mmHg)	185/110	110/72	114/61
Temperature (degrees Celsius)	37.2	37.8	36.4
Physical examination findings on admission	Intubated, rhonchi at bilateral lungs	Tachypnea; oxygen saturation 88% on room air	Intubated, rhonchi at bilateral lungs
COVID-19 PCR test	Positive	Positive	Positive
WBC count (×10^9^)	15.9	19.8	12.5
PLT count (×1,000 cells/μl)	567	239	218
ALT (U/L)	65	72	29
AST (U/L)	55	79	36
Creatinine (mg/dl)	0.63	1	4.33
Troponin (pg/ml)	<0.012	<0.012	<0.012
Lactic acid (mmol/L)	1.2	1.5	1.4
Ferritin (ng/ml)	695	710	1,160
C reactive protein (mg/dl)	4.9	26.7	7.5
LDH (Units/L)	461	475	824
D-dimer (ng/ml)	5,250	722	4,065
Partial thromboplastin time (s)	28.3	27.7	54.3
Prothrombin time (s)	10.5	18.8	17.3
Total bilirubin (mg/dl)	0.8	1.1	0.7
Alkaline phosphatase (Units/L)	257	136	61

## Hospital Course

Patient 1 was a 35-year-old African American male with past medical history (PMH) of hypertension and type 2 diabetes presented to a local emergency department (ED) with 3 days of fatigue, fever, abdominal pain, and cough. He was initially diagnosed with diabetic ketoacidosis after missing his medications for several days in the setting of bilateral pneumonia. He was eventually diagnosed with COVID-19 by polymerase chain reaction (PCR) test and received treatment with convalescent plasma, remdesivir, and dexamethasone. The patient continued to clinically decline with progressive hypoxia and was intubated and placed on mechanical ventilation due to severe ARDS. Patient was transferred to current facility after intubation. On admission, patient was intubated, and the auscultation of lungs revealed bilateral rhonchi. Soon after, ECMO was initiated, and he was started on continuous heparin infusion to prevent thromboembolic events. The patient's platelet level was above normal (567,000 cells/μl) range and gradually decreased to 253,000 cells/μl on the second day of heparin treatment. Venous ultrasound identified upper and lower extremity deep venous thrombosis (DVT) on hospital day 3, involving the right axillary, brachial veins, left subclavian veins, and axillary veins. Clots were not noticed in the ECMO circuit. Given a decreasing platelet count and slightly elevated heparin antibody level, the medical team suspected HIT with thrombosis. Heparin infusion was stopped and argatroban infusion was initiated. Despite this change, the platelet level did not improve, and the serotonin release assay eventually was found to be negative, indicating that HIT was unlikely. Argatroban was switched to bivalirudin. The patient did not develop active bleeding. In this case, the patient's thromboembolism was likely related to COVID-19-induced hypercoagulability, combined with the known risk of thromboembolism in patients receiving ECMO. The patient had been on ECMO for 52 days and expired due to asystole during hospitalization on hospital day 53 (Table 3).

**Table 3:** Thrombocytopenia and thromboembolic events on ECMO support.

Platelet on admission (cells/μl)	567,000	239,000	218,000
ECMO type	V-V	V-V	V-V
Time of ECMO initiation since admission	Day 2	Day 5	Day 2
Platelet level on first day of ECMO (cells/μl)	462,000	153,000	177,000
Initiation of anticoagulation	Day 1	Day 1	Day 1
Time of development of thrombocytopenia since admission	Day 30	Day 7	Day 34
Platelet nadir (cells/μl)	52,000	61,000	30,000
Time of thromboembolism development since admission	Day 3	Day 5	Day 3
Type of thromboembolism	Multiple DVT, diagnoses by venous ultrasound	PE, diagnosed by chest CTA	Multiple DVT, diagnosed by venous ultrasound
Hemorrhage requiring transfusion	Yes (day 26)	Yes (day 44)	No
Site of bleeding	Gastrointestinal tract	Hemothorax	None
ECMO circuit thrombosis	No	No	No
HIT	No	No	No
Stroke	No	No	No
Total ECMO days	52	23	22
Hospital days of stay	53	58	38
Outcome	Deceased	Discharged	Discharged
Cause of death	Asystole	None	None

Patient 2 was a 51-year-old African American male with a history of hypertension and type 2 diabetes presented to the ED for dyspnea and fever and was diagnosed with COVID-19 pneumonia by PCR test. Physical examination on admission revealed tachypnea with clear lung auscultation. Patient's oxygen saturation was 88% on room air. His hospitalization course was complicated by acute hypoxemic respiratory failure and septic shock. ECMO was initiated on hospital day 4 along with continuous heparin infusion for thromboprophylaxis. The patient's platelet level was within normal range on admission then decreased to 129,000 cells/μl on hospital day 6 at which time he was found to have a PE by chest computed tomography angiography (CTA). No clots were found in the ECMO circuit. Considering possibility of HIT, heparin was switched to argatroban on day 8. Heparin antibody was mildly elevated with negative serotonin release assay result making the diagnosis of HIT unlikely. He continued on argatroban, and with time, his clinical symptoms improved and his platelet level rose to normal range. He was managed on ECMO for 23 days and discharged to long-term care facility on hospital day 58.

Patient 3 was a 52-year-old Hispanic male with no significant PMH presented at a local ED for dyspnea and fever. He was diagnosed as having COVID-19 pneumonia by PCR test. The patient was transferred to current facility because of acute hypoxemic respiratory failure. On admission, patient was intubated, and physical examination revealed bilateral pulmonary rhonchi. ECMO was initiated on hospital day 2. Heparin was started for thromboprophylaxis. His platelet level was normal on admission. He suffered progressive thrombocytopenia on ECMO, and on hospital day 3, venous ultrasound revealed partially occlusive DVT in the distal left femoral vein, left popliteal vein, and left posterior tibial vein and occlusive DVT in the right subclavian vein, axillary vein, and brachial vein. Given concern for HIT, bivalirudin was used for anticoagulation to treat DVT and for thromboprophylaxis on ECMO. Serotonin release assay was negative, indicating that HIT was unlikely. Clots were not identified in the ECMO circuit. Heparin was reinitiated after ECMO decannulation on hospital day 24. His course was complicated by thrombocytopenia again (36,000 cells/μl) in the setting of sepsis secondary to intra-abdominal infection due to malpositioned percutaneous endoscopic gastrostomy (PEG) tube and delivery of tube feedings to peritoneum. Given at least intermediate risk for HIT at the time of re-exposure, heparin was again switched to bivalirudin. Heparin antibody level was borderline positive, and serotonin release assay result was again negative. His infection was well-controlled with antibiotics, and his COVID testing returned persistently negative results. However, the patient's platelet level did not improve, and he developed chronic respiratory failure. No active bleeding was identified during the hospitalization course. Warfarin was recommended as a long-term anticoagulant, considering his renal function and the patient was discharged to long-term care facility.

## Discussion

As we know, ECMO increases the incidence of thrombotic events (10), and COVID-19 patients are at high risk to develop thromboembolism. A recent study discussed the hemorrhagic and thrombotic events on eight COVID-19 patients who were managed with ECMO (12). Incidences of oxygenator thrombus, trachea hemorrhage, and oronasal hemorrhage were high among the eight patients. Similar findings were not observed in our retrospective study. However, there were several limitations of our study. Our study emphasized on the hematological complications including thrombocytopenia, thromboembolism, and anticoagulation management on ECMO-treated COVID-19 patients. The sample size of our study was small and may not represent the general population. Biases may present in the retrospective, single-center observational study. ECMO initiation criteria and anticoagulation management guidelines may vary among the different hospital facilities.

The coexistence of thrombocytopenia and thromboembolism in COVID-19 patients should raise physicians' concern. Studies suggest that thrombocytopenia is caused by COVID-19-induced bone marrow suppression and platelet consumption and destruction (1, 2). Cytokine storm and IL-6 production triggered by COVID-19 infection play a prominent role in the development of thromboembolism by activating coagulation pathway and promoting synthesis of coagulating factors. The specific virus mechanism is also involved in the process of thromboembolism. COVID-19 induces activation of renin-angiotensin system and amplifies the production of angiotensin. Through its prothrombotic and pro-inflammatory effects, angiotensin contributes to thromboembolic events including DVT and PE (13).

Venous thromboembolism prophylaxis is indicated in all hospitalized COVID-19 patients unless the risks of bleeding outweigh the benefit of prophylaxis (14), especially those who are treated with ECMO. Early identification and initiation of therapeutic anticoagulation treatment play an essential role in improving the outcome. Dynamic monitoring of D-dimer and platelet level provides valuable assessment of thrombotic events. Thrombocytopenia has been reported in both ECMO-treated patients and COVID-19 patients. It is associated with high mortality among hospitalized COVID-19 patients (15). Although thrombocytopenia increases the risk of active bleeding, incidence of bleeding in COVID-19 infection was lower compared with other viruses such as Ebola virus (16). The possible rationale is that COVID-19 coagulopathy leads more toward a hypercoagulable inflammatory state overcoming bleeding risk due to thrombocytopenia (16). Recent studies suggest that steroids, intravenous immune globulin (IVIG), thrombopoietin receptor agonists (TPO-RA), and platelet transfusion are all options to treat severe thrombocytopenia with active bleeding (17). Steroids seem to be a good initial choice if there are no contraindications. One concern considering the use of TPO-RA in patients with COVID-19 pneumonia is increased medication-induced thrombotic potential in patients who already have a prothrombotic state due to COVID-19 infection. IVIG may be initiated to immediately elevate platelet level and is reserved as the second-line treatment. Platelet transfusion can be used in those refractory to IVIG and especially for those with life-threatening bleeding (18). All choices should be carefully considered, and treatment plans individualized for each patient.

In conclusion, in the setting of coexistence of thrombocytopenia and thromboembolism, all hospitalized COVID-19 patients should be on thromboembolic prophylaxis, especially those who are treated with ECMO. Although those patients are at higher risk of bleeding due to low platelet level, treatment of thrombocytopenia should not be initiated unless there is active bleeding or platelet level is lower than 30,000 cells/μl. Anticoagulants should be held if platelet level is <30,000 cells/μl (18). Further investigation into the implications and mechanisms of thrombocytopenia and venous thromboembolism in patients with COVID-19 pneumonia will lead to better outcomes for our patients.

## Data Availability Statement

The original contributions presented in the study are included in the article/supplementary material, further inquiries can be directed to the corresponding author/s.

## Ethics Statement

The studies involving human participants were reviewed and approved by HCA Health Care. The patients/participants provided their written informed consent to participate in this study. Written informed consent was obtained from the individual(s) for the publication of any potentially identifiable images or data included in this article.

## Author Contributions

All authors listed have made a substantial, direct and intellectual contribution to the work, and approved it for publication.

## Author Disclaimer

The views expressed in this publication represent those of the author(s) and do not necessarily represent the official views of HCA Healthcare or any of its affiliated entities.

## Conflict of Interest

The authors declare that the research was conducted in the absence of any commercial or financial relationships that could be construed as a potential conflict of interest.

## Publisher's Note

All claims expressed in this article are solely those of the authors and do not necessarily represent those of their affiliated organizations, or those of the publisher, the editors and the reviewers. Any product that may be evaluated in this article, or claim that may be made by its manufacturer, is not guaranteed or endorsed by the publisher.

## Abbreviations

ARDS: acute respiratory distress syndrome
COVID-19: coronavirus disease 2019
CTA: computed tomography angiography
DVT: deep venous thrombosis
ECMO: extracorporeal membrane oxygenation
ED: emergency department
HIT: heparin induced thrombocytopenia
IL-6: interleukin 6
IVIG: intravenous immune globulin
PE: pulmonary embolism
PCR: polymerase chain reaction
PEG: percutaneous endoscopic gastrostomy
PMH: past medical history
TPO-RA: thrombopoietin receptor agonists.
